# Utilizing quantitative dried blood spot analysis to objectively assess adherence to cardiovascular pharmacotherapy among patients at Kenyatta National Hospital, Nairobi, Kenya

**DOI:** 10.1371/journal.pone.0280137

**Published:** 2023-01-20

**Authors:** David Wata, John Ogwu, Louise Dunford, Graham Lawson, Sangeeta Tanna

**Affiliations:** 1 Department of Pharmacy, Kenyatta National Hospital, Nairobi, Kenya; 2 Faculty of Health and Life Sciences, Leicester School of Pharmacy, De Montfort University, Leicester, United Kingdom; 3 Faculty of Health and Life Sciences, Leicester School of Allied Health, De Montfort University, Leicester, United Kingdom; University of Colorado Denver Skaggs School of Pharmacy and Pharmaceutical Sciences, UNITED STATES

## Abstract

The burden of cardiovascular disease (CVD) is rising in Kenya and non-adherence to cardiovascular pharmacotherapy is a growing global public health issue that leads to treatment failure, an increased risk of cardiac events and poor clinical outcomes. This study assessed adherence to selected cardiovascular therapy medications among CVD patients attending outpatient clinics at Kenyatta National Hospital, Kenya by determining drug concentration(s) in patient dried blood spot (DBS) samples. Patients who had been taking one or more of the five commonly prescribed CVD medications (amlodipine, atenolol, atorvastatin, losartan, and valsartan) for at least six months were enrolled. Each patient completed a short questionnaire about their medication history and then provided a finger-prick blood spot sample from which drug concentrations were determined by liquid chromatography-high resolution mass spectrometry analysis. Two hundred and thirty-nine patients (62.3% female) participated in the study. The median number of medications used by patients was 2 (IQR 75%-25% is 3–1). Less than half (117; 49.0%) of patients were adherent to their prescribed CVD pharmacotherapy. Binary regression analysis revealed a significant correlation between non-adherence and the number of medications in the treatment regimen (Odds Ratio (OR) 1.583; 95%CI: 0.949–2.639; P-value = 0.039) and that gender was not an independent predictor of medication adherence (OR 1.233; 95%CI: 0.730–2.083; P-value = 0.216). Valuable information about adherence to each medication in the patient’s treatment regimen was obtained using quantitative DBS analysis showing that adherence to CVD medications was not uniform. DBS sampling, due its minimally invasive nature, convenience and ease of transport is a useful alternative matrix to monitor adherence to pharmacotherapies objectively, when combined with hyphenated mass spectrometry analytical techniques. This information can provide physicians with an evidence-based novel approach towards personalization and optimization of CVD pharmacotherapy and implementing interventions in the Kenyan population, thereby improving clinical outcomes.

## Introduction

Cardiovascular diseases (CVD) are the leading cause of morbidity and mortality globally accounting for 17.9 million deaths per year in 2019 representing 32% of total deaths. These non-communicable diseases (NCD) are a group of disorders of the heart and blood vessels and include coronary heart disease, cerebrovascular disease and ischemic heart attacks. Over 75% of CVD deaths take place in low- and middle-income countries (LMIC) including Kenya [1]. In Kenya the burden of CVD and their risk factors is significant and escalating and account for 25% of hospital admissions and 13% of all deaths [2]. According to the Kenya Health Policy 2014–2030 CVD are a major public health concern with significant economic implications for Kenya in terms of healthcare needs, lost productivity and premature death thus placing a heavy burden on the national economy and reducing Kenya economic growth [3].

The prevention, treatment and management of CVD plus associated risk factors, including hypertension and dyslipidemia, involves the long-term use of medications. A combination of medications is often employed and these include antihypertensives, hypolipidemic drugs, diuretics, anticoagulants and antiplatelet drugs. The most commonly prescribed CVD drugs in Kenya include amlodipine, atenolol, atorvastatin, bisoprolol, enalapril, hydrochlorothiazide, losartan and ramipril [4]. An essential factor in managing and treating CVD properly is to ensure CVD patients adhere to their prescribed pharmacotherapy and thus take their medication(s) as recommended and continually. Adherence to medications by patients helps to ensure that their blood drug(s) concentration(s) are within the therapeutic limits in order to maximize their effectiveness. Medication adherence is defined by the World Health Organization (WHO) as the ability of a patient to take their medications in the way recommended by their healthcare provider, and the failure to do so is termed non-adherence [5]. Non-adherence to medications is an understated public health problem worldwide and a major issue in situations where self-administration of oral medications is required [6] and in circumstances where polypharmacy and complex drug regimens exist [7,8]. It is documented that as many as 50% of CVD medications are not taken by patients as prescribed [9–11]. This suboptimal adherence to CVD pharmacotherapies leads to poor health consequences for patients and is associated with a substantial increase in CVD events [12]. Additionally, it negatively impacts on national healthcare systems because it reduces the effectiveness of the pharmacological treatment and is associated with avoidable morbidity and mortality, medicines wastage, increased hospital admissions and higher costs of care [9,13–15]. In low resource countries including Kenya, the pervasive threat of poor-quality falsified and substandard pharmaceutical products in supply chains is likely to exacerbate this public health problem by giving rise to unintentional non-adherence when patients take such poor-quality medicines unknowingly [6]. Given the high human and economic cost linked to non-adherence to CVD drug therapies the accurate assessment of adherence is a vital action to ensure that physicians make informed pharmaceutical care decisions and that patients derive the full benefits of their prescribed pharmacotherapies and reduce medicines wastage and costs for Kenya healthcare providers.

Currently, there is no “gold standard” method for assessing medication adherence in routine clinical pharmacy practice but a variety of strategies have been tested since each strategy has its advantages and limitations [6,16]. These include indirect strategies such as pill counts, prescription refill rates, patient diaries or self-reports, questionnaires and clinical markers as well as direct strategies including bioanalytical tests that involve the measurement of urine or blood plasma/serum drug or metabolite levels. Indirect methods are easy to use, quick and low cost but cannot confirm if the patient has taken their medication correctly and are proxy measures of medication adherence [17]. For instance, pill counts simply confirm the number of tablets removed from their original container but cannot corroborate if these tablets have actually been consumed by the patient. Although questionnaire tools are widely used, they are subjective as the evaluation is dependent on the participant’s belief and recall bias [18]. Furthermore, this indirect adherence assessment strategy provides no information about the time a dose was taken and is unable to take into consideration drug pharmacokinetics and pharmacodynamics which vary from patient to patient and which may be fundamental in establishing clinical outcomes [6]. Direct assessment approaches are more accurate methods of assessing adherence to pharmacotherapy. Current measures are, however, costlier in terms of both patient and physician time and acquiring liquid blood samples requires a visit to a hospital or clinic. Urine is a common choice of biological sample matrix as the collection of urine samples is non-invasive. Storage and shipping costs of urine samples can be prohibitive particularly if the bioanalytical testing laboratory is overseas. Furthermore, some patient groups may be reluctant to provide this biosample due to religious, cultural or ethical issues [19]. Tanna and Lawson proposed that the costs associated with direct assessment can be reduced without detriment to the information produced, by use of a fingerpick microvolume blood sample collected as a dried blood spot (DBS) for the determination of drug as a measure of medication adherence [19–21]. The ease of sample collection, storage and transport in addition to presenting a low biohazard risk offered by microsampling methods such as DBS mean that it is a feasible, practical and sustainable option to objectively assess medication adherence especially remotely and in resource-limited settings [19,22]. This microsampling approach also eliminates the need of phlebotomy thereby enabling self and/or remote collection of the DBS [23]. In Kenya, to date, the assessment of adherence to antihypertensives has been conducted using questionnaires [24,25], patient self-reports and pill counts [26,27] and clinical marker monitoring [26] and these investigations have revealed adherence to these CVD medications ranges from 67% to 94% [24–27].

The aim of this study was to assess Kenyan patients’ adherence to treatment with amlodipine, atenolol, atorvastatin, losartan and valsartan by the quantification of these target CVD medications in DBS samples collected from the patients. Kenyatta National Hospital (KNH), Nairobi was selected as the study site since it is Kenya’s largest public referral and teaching hospital which serves patients who are mostly referred from peripheral sites (counties) and often treats patients who are some of the poorest and vulnerable members of society and where non-adherence to CVD medications is likely to be a burgeoning public health issue.

## Materials and methods

### Study setting, design and ethical approval

This study was conducted at KNH, a level 6 teaching and referral public health facility in Nairobi, Kenya. The target study sample size was 270 and the sample size calculation was determined using the Daniel equation based on the assumption that the CVD prevalence in Kenya is approximately 22.8% [28]. In Kenya ethical approval for the study was obtained from the Kenyatta National Hospital-University of Nairobi Ethics and Research Committee (Reference: P298/04/2019). In the UK ethical approval was granted by De Montfort University’s Faculty of Health and Life Science Research Ethics Committee (Reference: 1747). Both informed and written consent was sought from each participant using a consent form prior to enrollment in the study.

### Inclusivity in global research

Additional information regarding the ethical, cultural, and scientific considerations specific to inclusivity in global research is included in the Supporting Information (S1 File).

### Participants

Participants were recruited from the general medicine, cardiology and endocrine outpatient clinics at KNH during a routine clinical visit between the study period of January 2020 and March 2020. Eligible participants were patients aged ≥18 years who had been prescribed one or more of the target CVD medications for more than six months prior to recruitment, had no visual or cognitive impairments and able to understand and communicate in English. The target CVD medications were amlodipine, atenolol, atorvastatin, losartan and valsartan. Pregnant women, non-English speakers, and participants that required admission were excluded from the study. The hospital pharmacy department personnel screened patients during the study period by applying the selection criteria when attending their appointment and assessed for eligibility. If eligible, the patients were invited to participate. The KNH pharmacy department personnel then re-checked the eligibility and those who agreed to participate were provided information about the study by the pharmacy personnel who gave each patient an English participant information leaflet and obtained written informed consent. For each participant adherence to prescribed cardiovascular therapies was assessed by collection of a simple finger-prick blood sample on a DBS collection card at KNH and its subsequent liquid chromatography-high resolution mass spectrometry (LC-HRMS) analysis at De Montfort University, UK.

### Collection of patient blood spot samples and baseline medication information

Prior to collection of the DBS samples each study participant was requested to supply baseline medication data on the number and name of prescribed CVD medicine(s), dose, dose frequency, approximate time since last dose and name of other prescribed medication(s). This was via a mini CVD drug prescription questionnaire in English. This crucial baseline information together with CVD drug pharmacokinetic information allowed the research team to establish if the analysed blood drug concentration(s) was within the therapeutic window and if the patient had been adherent to their prescribed pharmacotherapy [19,21]. In addition to this, the baseline medication data allowed the research team to ascertain the total number of prescribed medications in a patient’s treatment regimen.

Participant DBS samples were obtained by a simple fingerprick using a sterile lancet and 2 or 3 drops of blood were collected on a Whatman 903 DBS sample collection card (GE Healthcare). Each DBS sample collection card was then allowed to dry at room temperature for 2–3 hours. After drying each card was stored in an individual reference number labelled plastic re-sealable bag, containing desiccant, and shipped to De Montfort University, UK under ambient conditions for LC-HRMS analysis.

### Extraction and analysis of patient DBS samples

Solvent extraction of the target analytes from the patient DBS samples was carried out using the protocol detailed in our previously published work [29,30]. The concentrations of amlodipine, atenolol, atorvastatin, losartan and valsartan in DBS extracts were determined using a previously validated LC-HRMS bioanalytical assay on an Agilent 1290 LC coupled with an Agilent G6530A QTOF mass spectrometer [29,30].

### Adherence assessment based on the LC-HRMS analysis of DBS samples

Patients were considered non-adherent by quantitative DBS analysis when one or more of their prescribed CVD medication concentrations were non-detectable or < 5.25% of published C_max_ or > published C_max_ [19,21].

### Statistical analysis

Data are presented as frequencies/percentages or medians with 25%-75% interquartile range (IQR). Binary logistic regression analysis was used to examine the relationship between non-adherence as assessed by quantitative DBS analysis and gender and the number of different medications prescribed to a patient. For the purposes of binary regression analysis, the study participants were divided into two categories, i.e. non-adherent or adherent. Mean and standard deviation values were used to express the concentrations of each target CVD medication in the DBS samples for individual patients.

## Results and discussion

### Patient characteristics

Two hundred and forty-five participants were recruited and from this, six participants had incomplete information and were excluded from the study. Two hundred and thirty-nine participants fulfilled the inclusion criteria and of these 62.3% were female and 37.7% were male. The median number of medications per regimen was 2 and 50.2% of the participants were prescribed >2 medications (Table 1). Data on the CVD medications prescribed to patients in the KNH cohort are presented in Table 2. In this cohort 5.9% of the patients were prescribed a β-blocker, 60.7% angiotensin II receptor blockers, 60.3% a statin and 42.7% a calcium channel blocker. Atorvastatin (60.3%) and losartan (60.3%) were the most widely prescribed CVD medications followed by amlodipine (42.7%). Atenolol (5.9%) and valsartan (0.4%) were the least prescribed medications in this KNH patient cohort.

**Table 1:** Patient demographics.

Demographic variables	Total study population (N = 239)	
	N	%
**Gender**		
Female	149	62.3
Male	90	37.7
**Number of medications**		
1–2	119	49.8
3–4	108	45.2
5–6	11	4.6
>6	1	0.4
Median (IQR) = 2 (3–1) Q1 (25%) = 1 Q2 (50%) = 2 Q3 (75%) = 3		

IQR: Interquartile range.

**Table 2:** Breakdown of prescribed CVD medications in the Kenyatta National Hospital cohort.

Medication type	N (%) of patients
**β blocker**	
Atenolol	14 (5.9)
**Angiotensin II receptor blockers**	
Valsartan	1 (0.4)
Losartan	144 (60.3)
**Statin**	
Atorvastatin	144 (60.3)
**Calcium channel blocker**	
Amlodipine	102 (42.7)
**Total number of participants**	239 (100)

### Adherence assessed using drug concentrations determined in patient DBS samples

The KNH patients were categorized as adherent and non-adherent on the basis of their blood CVD drug concentrations determined by LC-HRMS analyses. Medication adherence is indicated by a blood drug concentration between the published C_max_ concentration and 5.25% of C_max_ i.e. the drug concentration after 5 half-lives, when it is considered to be therapeutically inactive. Conversely, medication non-adherence is denoted by the absence of the drug in the volunteer’s DBS sample or if the drug level determined is outside its therapeutic window [19,21]. The patient DBS samples collected at KNH were shipped to De Montfort University, UK for LC-HRMS analyses.

All of the target CVD drugs in the DBS samples collected from the KNH patients were considered stable since all DBS samples were analysed within the validated stability period for this bioanalytical assay [29,30]. Furthermore, in the LC-HRMS analyses of the DBS samples all target CVD drugs from the KNH patient DBS samples plus quality control DBS samples within each analytical run were within 15% of their baseline concentration and therefore considered stable.

The assessment of adherence by determination of the target drug concentration by LC-HRMS analyses of KNH patient DBS samples found that only 117 (70 female:47 male) KNH patients (49.0%) were adherent to their prescribed CVD medications and 122 (79 female:43 male) patients (51.0%) were non-adherent (Table 3). This level of adherence is in line with the 50% figure reported by the WHO for adherence to medications for chronic illnesses in developed countries [5].

**Table 3:** Binary logistic regression analysis identifying factors predicting medication adherence.

Parameter	Adherent N (%)	Non-adherent N (%)	Odds Ratio (OR)	95% CI for OR Lower-Upper	P-value
**Gender**					
Male	47 (52.2)	43 (47.8)	1		
Female	70 (47.0)	79 (53.0)	1.234	0.730–2.083	0.216
**No. of medications**					
1–2	68 (54.4)	57 (45.6)	1		
>2	49 (32.2)	65 (67.8)	1.583	0.949–2.639	0.039*

**Note**:

*P<0.05.

This is the first study conducted in Kenya to gain knowledge about adherence to CVD medications in Kenya using a direct and objective bioanalytical testing method. Previous medication adherence assessment studies in Kenya used indirect methods only [24–27] thus, there is no previous data about the level of non-adherence found by direct methods to compare with in Kenya. The level of medication adherence reported in this study is not concordant and considerably lower than those reported using the indirect and subjective measures of medication adherence in Kenya previously [24–27]. This is likely to be attributed to the fact that indirect measures are subject to overestimation of adherence. The study findings are in line with a previous similar study conducted in Iraq which reported 50.8% adherence to CVD medicines assessed using a DBS bioanalytical assay [18]. The objective DBS-based LC-HRMS bioanalytical assay employed in the current study can determine the concentrations of the five target cardiovascular medications in one analytical run, which represents cost-effectiveness for KNH by helping to identify non-adherent patients quickly and by helping to decrease patient spending on health and facilitating medicines optimization within clinical practice.

The objective medication adherence assessment strategy employed in this study showed that gender was not significantly associated with medication non-adherence (OR 1.234; 95% CI: 0.730–2.083; P-value = 0.216) (Table 3). Conflicting results on the correlation between adherence to CVD pharmacotherapy and gender are reported in the literature thus suggesting that complex sociological gender-based dynamics coupled with behavioral factors are at play [31].

Medication adherence assessed using drug concentration in the microvolume DBS samples showed a positive correlation between non-adherence and the number of medications in the patient’s treatment regimen (OR 1.583; 95% CI: 0.949–2.639; P-value = 0.039) (Table 3). These findings are in line with previous studies which show that the number of prescribed medications coupled with regimen complexity influence adherence to pharmacological treatments [18,32,33]. Polypharmacy is common practice to treat and manage CVD and to improve mortality and morbidity, however it is a known contributor to intentional medication non-adherence [34] and can expose patients to increased risk of adverse drug reactions and drug-drug interactions [35]. Fixed dose combination therapy of CVD medications may reduce the pill burden on patients and offer a convenient and effective approach improving CVD pharmacotherapy adherence and to manage CVD risk factors [36].

Table 4 gives the breakdown of adherence and non-adherence to each target CVD drug amongst the study participants using the objective patient microsample analysis data. In Table 4 the number of non-adherent patients were classified into three categories analogous to a previous study conducted in an Iraqi patient cohort [18]. These are: (i) the number of patients with no detectable drug in their DBS sample i.e. the concentration of target CVD drug which falls below the limit of quantification (LOQ) of the LC-HRMS bioanalytical assay; (ii) the number of patients with target drug concentration in between the LOQ and 5.25% of the target drug C_max_; (iii) the number of patients with target drug concentrations >C_max_.

**Table 4:** Adherence and non-adherence amongst the Kenyan CVD patient cohort assessed using quantitative dried blood spot analysis of patient samples by LC-HRMS.

CVD medication	No. of patients	LOQ of LC-HRMS assay (ng/ml)	No. of adherent patients (%) Drug concentration in between 5.25% of C_max_ and < C_max_	No. of non-adherent patients (%)		
				(i) Patients with no detectable drug concentration <LOQ	(ii) Patients with drug concentration in between the LOQ and 5.25% of C_max_	(iii) Patients with drug concentration > C_max_
**Amlodipine**	102	0.5	66(64.7)	32(31.4)	-	4(3.9)
**Atenolol**	14	10	9(64.3)	-	-	5(35.7)
**Atorvastatin**	144	0.5	74(51.4)	69(47.9)	-	1(0.7)
**Losartan**	144	5	95(66.0)	45(31.3)	-	4(2.7)
**Valsartan**	1	50	1(100)	-	-	-

Table 4 shows that the majority of the non-adherent patients fell into category (i) where the LC-HRMS bioanalytical assay was unable to quantify amlodipine, atorvastatin or losartan in those patient DBS samples. In comparison the least number of patients fell into category (iii) where their concentrations of amlodipine, atorvastatin, atenolol or losartan exceeded the drug C_max_. This latter result is likely to indicate that the incorrect medication dose had been taken by these patients or an error in the dosage regimen, or a slower rate of drug elimination, or a decreased renal and hepatic function leading to drug accumulation or drug-drug interactions and thus warrant further investigation. Zero patients fell into category (ii) which would be indicative of patients who had skipped a dose of the target CVD drug prior to DBS sample collection or patients who had taken their medication a few hours before their hospital visit. Table 5 gives a further breakdown on the category (i) non-adherent patients who had drug concentrations falling below the LOQ of the LC-HRMS bioanalytical assay.

**Table 5:** Breakdown of non-adherent patients due to no detectable CVD drug concentration <LOQ of LC-HRMS-based dried blood spot bioanalytical assay.

CVD medication	LOQ of LC-HRMS assay (ng/ml)	No. of non-adherent patients due to (i) no detectable drug concentration <LOQ (%)	
		Medication taken in 24 hours	Medication taken in > 48 hours
**Amlodipine**	0.5	27 (84.4)	5 (15.6)
**Atenolol**	10	-	-
**Atorvastatin**	0.5	53 (76.8)	16 (23.2)
**Losartan**	5	39 (86.7)	6 (13.3)
**Valsartan**	50	-	-

Table 5 shows that the non-adherent patients categorized due to absence of drug in their blood could be categorized further into patients who had ingested their prescribed CVD medication tablet as recommended and patients who had clearly been non-adherent and had not taken the medication for over 48 hours. The baseline medication questionnaires for the latter patients revealed that they had not taken their medications for weeks or months. Unsurprisingly, an objective bioanalytical test for assessing medication adherence for such patients would result in non-detectable drug levels as was the case in this investigation. Discussions with these patients must be initiated to investigate the reasons for non-adherence. Since non-adherence to prescribed CVD pharmacotherapy is associated with increased cardiac events and further complications it is crucial to educate patients about the importance of medication adherence. Pharmacy personnel in Kenya can play a very important role in educating the patients about their diseases and medication in addition to providing key information about medication-taking behavior to physicians so that they can make an informed decision about future treatment. In this study if the patient baseline data revealed that the patient had taken their medication as prescribed but the subsequent LC-HRMS analyses showed non-detectable drug in that patient’s DBS sample then this suggests that the patient is likely to have unknowingly ingested a substandard or falsified pharmaceutical product and was therefore unintentionally non-adherent. The circulation of poor-quality medicines in the Kenyan market is documented [37–39] and the prevalence ranges from 14–38% [38,40]. It is postulated that if a patient ingests such poor-quality medicines their blood drug levels will not reach the required therapeutic levels since such medications contain little no active pharmaceutical ingredient and this can lead to treatment failure [6]. This study provides a pointer to this growing public health issue in Kenya and thus warrants investigation and action by Kenya medicine’s regulatory authority and healthcare system policymakers to implement adequate pharmacovigilance policies to achieve desired patient health outcomes from adhering to prescribed pharmacotherapy.

Table 5 also shows that 27 patients had clearly not taken their medications as recommended as these resulted in non-detectable concentrations in the objective DBS analysis test. Forgetfulness, medication side effects and access to medicines could all account for this non-adherence and KNH healthcare professionals should now investigate the main reasons why this cohort did not take their CVD treatment. Another reason why patients may not take their prescribed medications is due to stock outs at KNH. If a particular medication is not available at the KNH pharmacies, then the patient may altogether not take this medication. If a patient finds the medication to be costly for them, they also will not purchase the medicine and thus will not take it. Reports by patients not purchasing medications due to cost is not uncommon in KNH. It is reported that essential CVD medicines are unavailable and unaffordable for a large proportion of communities where the individuals with high risk of CVD are living, particularly in LMICs such as Kenya [41,42]. The unavailability and unaffordability of essential CVD medicines is associated with increased risk of cardiac events and a worsening of the patient’s health. In Kenya, stock outs of essential medications in public healthcare facilities is an important factor which may contribute to the patient unknowingly purchasing poor-quality CVD medications from unlicensed and illegal medicine outlets or unregulated websites. This therefore warrants further investigation in medicine price differences within different health sub-sectors in Kenya and between licensed pharmacies and unlicensed illegal medicine outlets [39].

**Fig 1:**
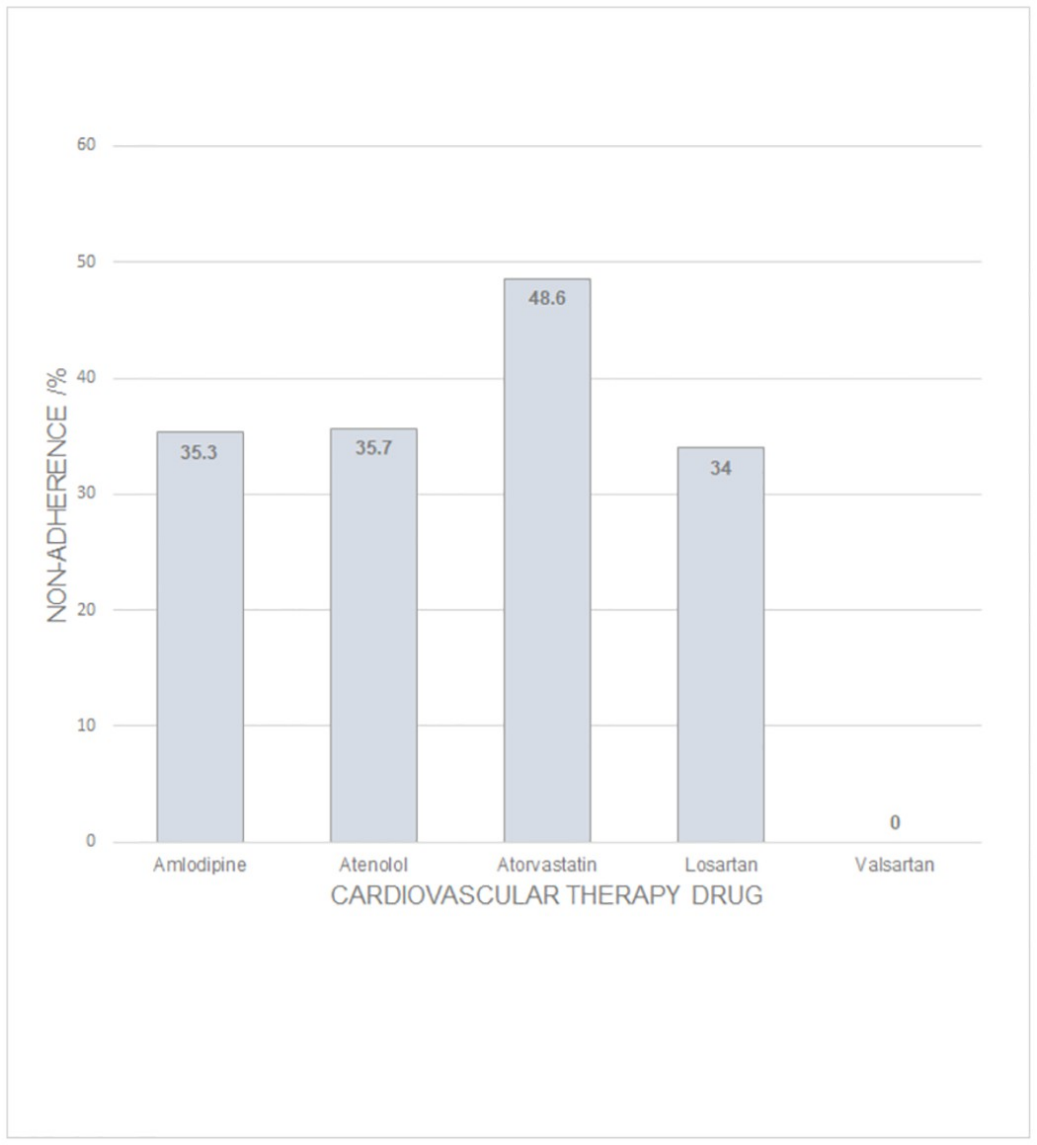
A comparison of non-adherence assessment to prescribed CVD pharmacotherapies using a quantitative dried blood spot-based LC-HRMS bioanalytical assay.

Table 4 and Fig 1 show that non-adherence to the target CVD medications in the KNH patient cohort, as assessed by quantitative DBS analysis is not uniform. Such differences would not be picked up if a questionnaire tool was employed to assess medication adherence. Whilst questionnaires are cheaper and easy to administer to patients they are unable to track adherence to each medication in a patient’s treatment regimen. Since polypharmacy is common in the treatment of CVD this is a drawback of using this type of indirect assessment method for assessing adherence to pharmacotherapy. Furthermore, when such indirect assessment strategies are employed it is not possible to track dosing error and/or prescription error or if the patient took the medication at the wrong time. Patients may take the wrong medication or the incorrect dose or at the wrong time. In these cases, whilst the medication-taking behavior exists the patient will not derive maximum therapeutic benefit from the ingested medication or may experience adverse side effects. Such indirect methods of assessment cannot also take into account patient-to-patient variation in pharmacokinetics, pharmacodynamics and pharmacogenetics, which affect drug concentrations in the blood. If a physician assumes that a patient is taking their prescribed medicine as recommended, he or she may attribute progression of the patient’s CVD condition to a lack of activity of the prescribed CVD drug and therefore may unnecessarily change a regimen [6]. This direct assessment strategy of using quantitative dried blood spot analysis is capable of providing accurate information about the drug concentrations of each drug and thus capable of tracking adherence to each medication in a treatment regimen. This objective information would be extremely helpful to physicians in terms of optimizing and individualizing pharmacotherapy for each patient.

Differences in the levels of adherence to prescribed CVD pharmacotherapy can be influenced by the medication-related side effects and medication class. It is reported that statins have lower adherence rates compared to other CVD medications [43]. This study supports these findings where the adherence to atorvastatin is 51.4% compared to 64.3% for atenolol, 64.7% for amlodipine and 66.0% for losartan. In Kenya, cardiovascular medicine availability, affordability, accessibility, acceptability and quality [38,40], in addition to retail price differences between generic and branded medicines [42], plus patient socioeconomic factors [44] may account for the differences observed in adherence levels for the target CVD drugs. Medicine price differences between the public and private health sub-sectors in Kenya could also influence changes in medicine availability [42].

The minimally invasive DBS microsample collection method used in this study offers advantages of ease of sampling and ease of storage for routine implementation in Kenya. A further benefit of this patient-friendly microsample collection method is that the DBS samples can be easily shipped for analyses to testing laboratories outside Kenya where advanced liquid chromatography-mass spectrometry-based instruments, required for DBS analyses and drug quantification, are readily available [45]. Fostering international collaborations will therefore go a long way in tackling the problem of medication non-adherence in LMIC countries such as Kenya.

## Conclusion

This is the first study to objectively assess adherence to prescribed CVD pharmacotherapy in Kenya using a direct assessment method of determining target drug concentrations in DBS samples. The study findings revealed that only 49.0% of the KNH CVD patients attending outpatient clinics were adherent to one or more of their prescribed CVD medications when assessed using the LC-HRMS analyses of patient DBS samples in this patient population.

The direct measurement of blood drug concentration(s) can provide objective information on the levels of each medication in the patient’s blood. In the event of poor patient progress, it is imperative the physician knows if the patient has followed the treatment regimen as prescribed and thus physicians would immensely benefit from an evidence-based framework to guide their pharmaceutical care decisions. The unambiguous medication-related nature of the blood drug concentration data in this study can help physicians to make informed decisions about future treatment on a personalized basis and to facilitate evidence-based discussions with the patient in order to understand the reasons for non-adherence.

The mortality rate associated with CVD in Kenya is very high and this may in part be due to non-adherence to CVD medications coupled with poor-quality medicines procured by patients. This study findings call for interventions and health policies to be put in place at a local and national level to address the public health issue and to achieve desired clinical outcomes for the Kenyan patients from the CVD drug therapies prescribed and to reduce Kenya healthcare provider costs and medicines wastage.

## Supporting information

S1 File Questionnaire on inclusivity in global research. (PDF)
